# Sweet complexity: *O*-linked protein glycosylation in pathogenic *Neisseria*

**DOI:** 10.3389/fcimb.2024.1407863

**Published:** 2024-05-14

**Authors:** Bente Børud, Michael Koomey

**Affiliations:** ^1^ Department of Bacteriology, Division for Infection Control and Environmental Health, Norwegian Institute of Public Health, Oslo, Norway; ^2^ Department of Biosciences, Section for Genetics and Evolutionary Biology, University of Oslo, Oslo, Norway

**Keywords:** immunogenicity, glycan diversity, immune escape, *Neisseria gonorrhoeae*, *Neisseria meningitidis*

## Abstract

The genus *Neisseria*, which colonizes mucosal surfaces, includes both commensal and pathogenic species that are exclusive to humans. The two pathogenic *Neisseria* species are closely related but cause quite different diseases, meningococcal sepsis and meningitis (*Neisseria meningitidis*) and sexually transmitted gonorrhea *(Neisseria gonorrhoeae*). Although obvious differences in bacterial niches and mechanisms for transmission exists, pathogenic *Neisseria* have high levels of conservation at the levels of nucleotide sequences, gene content and synteny. Species of *Neisseria* express broad-spectrum *O*-linked protein glycosylation where the glycoproteins are largely transmembrane proteins or lipoproteins localized on the cell surface or in the periplasm. There are diverse functions among the identified glycoproteins, for example type IV biogenesis proteins, proteins involved in antimicrobial resistance, as well as surface proteins that have been suggested as vaccine candidates. The most abundant glycoprotein, PilE, is the major subunit of pili which are an important colonization factor. The glycans attached can vary extensively due to phase variation of protein glycosylation (*pgl)* genes and polymorphic *pgl* gene content. The exact roles of glycosylation in *Neisseria* remains to be determined, but increasing evidence suggests that glycan variability can be a strategy to evade the human immune system. In addition, pathogenic and commensal *Neisseria* appear to have significant glycosylation differences. Here, the current knowledge and implications of protein glycosylation genes, glycan diversity, glycoproteins and immunogenicity in pathogenic *Neisseria* are summarized and discussed.

## 1 Introduction

Glycosylation is one of the most abundant and complex post translational modifications (PTMs) of living organisms. Bacteria have the ability to synthesize a variety of sugar structures such as capsular polysaccharides, lipooligosaccharides or lipopolysaccharides, peptidoglycans as well as *N*- and *O*-linked glycans of proteins. Understanding the biological significance of bacterial protein glycosylation has been hindered in part by the considerable diversity in structures and functions of glycans.

Early gas chromatographic analysis of *Neisseria gonorrhoeae* pili indicated the presence of 1–2 hexose groups per pilin subunit (Robertson et al., 1977). The pilin subunit PilE was later found to undergo *O*-linked glycosylation in both *Neisseria meningitidis* (Stimson et al., 1995) and *N. gonorrhoeae* (Parge et al., 1995). Further studies have shown that glycosylation has significant effects on the antigenicity and immunogenicity of the PilE protein (Børud et al., 2010). In 2009, with identification of eleven additional glycoproteins in *N. gonorrhoeae* (Vik et al., 2009) and AniA (aka NirK) in *N. meningitidis* (Ku et al., 2009), the first general *O*-linked protein glycosylation system in bacteria was described and is now one of the better characterized systems. In Gram negative bacteria, such *O*-linked protein glycosylation is characterized by the glycan being synthesized on an undecaprenyl pyrophosphate (Und-PP) lipid carrier in the cytoplasm, its subsequent flipping across the inner membrane to the periplasm and oligosaccharyltransferase (*O*-OTase) - mediated glycosylation of either serine or threonine residues of target proteins (Nothaft and Szymanski, 2010). In addition to *Neisseria*, *O*-linked protein glycosylation of this type has also been identified in *Burkholderia* (Lithgow et al., 2014), *Francisella* (Egge-Jacobsen et al., 2011), *Acinetobacter* (Iwashkiw et al., 2012), and the plant pathogen *Ralstonia solanacearum* (Elhenawy et al., 2016).

The genus *Neisseria* consist of Gram negative, oxidase-positive diplococci that are associated with mucosal surfaces. The two pathogenic *Neisseria* species are human restricted, substantial threats to global health and closely related, but cause very different diseases, meningococcal disease (*N. meningitidis*) and sexually transmitted gonorrhea (*N. gonorrhoeae*). *N. meningitidis* is most often a commensal bacterium with varying carriage prevalence (3–30%) in the human oropharynx (Trotter and Greenwood, 2007) that occasionally can cause invasive disease resulting in severe meningitis and/or septicemia. *N. gonorrhoeae* causes gonorrhea by primary colonization of the epithelium of the male urethra and female ectocervix and endocervix but can occasionally also cause pelvic inflammatory disease. The gonococcus can infect other mucous membranes in the genitourinary tract, rectum, oral cavity, pharynx, and eyes and might cause disseminated gonococcal infections. Despite their apparent differences, these pathogenic species are remarkably conserved and comprise a distinct clade in phylogenetic relationship to other *Neisseria* species (Bennett et al., 2012).

Studies of protein glycosylation have revealed that *Neisseria* species encompass numerous glycoproteins and display high intra- and interstrain glycoform variability (Ku et al., 2009; Vik et al., 2009; Børud et al., 2010, 2011; Anonsen et al., 2012b; Børud et al., 2014; Anonsen et al., 2017; Hadjineophytou et al., 2024). To date, it has been shown that one strain has the capacity to express between 7 and 15 different glycoforms by differing combinations of glycosyltransferases and the glycan *O*-acetylation (Anonsen et al., 2017; Wang et al., 2021). The complexity can be attributed to different molecular mechanisms and evolutionary events such as the *pgl* gene content, phase variable *pgl* genes, and allelic *pgl* variants where hypermorph, hypomorph, amorphic and even neomorph glycosyltransferases have been identified (Chamot-Rooke et al., 2007; Børud et al., 2011; Johannessen et al., 2012; Børud et al., 2014). In addition, *Neisseria* species display glycan microheterogeneity (different glycan structures at the same site of a protein) and macroheterogeneity (presence/absence of glycans at a particular residue).

## 2 Pilin is the major glycoprotein

*Neisseria* employ diverse mechanisms to alter the structure of their immune-exposed surface antigens. These involve hypervariable loci in their genomes; acting as diversity hotspots. This diversity provides a vast repertoire of epitopes for surface antigens and facilitate evasion of adaptive immune responses targeting specific variants. The type IV pilus colonization factor represents a major surface exposed antigen, and it is assembled in the periplasm and transported through the outer membrane by a complex involving over 20 different proteins. Type IV pili in *Neisseria* are homopolymers of the PilE protein that can extend several micrometers from the cell surface and are involved in initial attachment to epithelial and endothelial cells (Nassif et al., 1997), natural competence (Fussenegger et al., 1997) and twitching motility (Wolfgang et al., 1998).

A gonorrhea pilus vaccine was shown to be safe and antigenic, and it elicited a broad antibody response in serum and genital secretions that blocked attachment of gonococci to epithelial cells (Mcchesney et al., 1982). Disappointingly, a large-scale trial of the vaccine showed no evidence for protection; most probably due to pilus variation between the vaccine strain and the circulating strains (Boslego et al., 1991). The PilE pilin protein subunit is subject to high frequency, antigenic variation through gene conversion that results in dramatic changes in primary structure. At the time of the vaccine trial, it was not recognized that PilE was glycosylated. The neisserial pilins have been shown to have many different PTMs, including the glycan variants that will be further detailed here, but also phosphoform modifications such as phosphoethanolamine, phosphocholine, and phosphoglycerol (Stimson et al., 1996; Weiser et al., 1998; Hegge et al., 2004; Aas et al., 2006; Chamot-Rooke et al., 2011). The phosphoform modifications that are found near sites of glycan attachment might modulate pilin antigenicity and have the potential for dynamic interplay between the PTMs (Anonsen et al., 2012a). For instance, it has been suggested that pili modified with both glycan and phosphocholine is required for efficient meningococcal adherence to platelet activating factor receptor on human airway cells (Jen et al., 2013).

Two distinct classes of PilE have been identified where Class I pili are expressed by all *N. gonorrhoeae* isolates and certain *N. meningitidis* isolates, while Class II pili are found in the other *N. meningitidis* isolates. Class I pili exhibit high variability involving frequent gene conversion events via homologous recombination between the *pilE* locus and a series of inactive *pilS* silent, truncated gene copies (Hagblom et al., 1985; Zhang et al., 1992). In contrast to the situation for Class I pilin that carries a single glycosylated residue, *N. meningitidis* isolates that express antigenic invariable Class II pilin display multiple pilin glycosylation sites (Gault et al., 2015). Gault and colleagues hypothesized that *N. meningitidis* isolates that express antigenic invariable Class II pilins carry multiple pilin glycosylation sites to evade the immune system by fully masking protein epitopes (Gault et al., 2015).

In one earlier study, elimination of meningococcal pilin glycosylation was associated with modest increases in piliation levels and adherence to human epithelial cells (Virji et al., 1993; Marceau et al., 1998). It has also been suggested that gonococcal infection of the cervical epithelium requires the combined action of pilin, porin and iC3b (an opsonin and ligand for complement 3 receptor (CR3)) (Edwards et al., 2002), and that the pilin glycan is required for binding to iC3b and thus involved in activation of the CR3 (Jennings et al., 2011). PilE glycosylation has also been shown to impact on type IV pilin functions such as autoagglutination, the efficiency of pilin subunit polymerization, and the dynamics of pilus extension - retraction (Vik et al., 2012; Gault et al., 2015). In addition to PilE, several additional type IV biogenesis or related proteins (PilQ, PilN, PilH, PilI, PilJ, PilV, ComP) are glycosylated (Hadjineophytou et al., 2022, 2024) (**Table 1**). In contrast, the more distant commensal neisserial species have *O*-OTases that supported glycosylation of other proteins, but not pilin (Hadjineophytou et al., 2022). Interestingly, *Acinetobacter* have two functional *O*-OTases where one glycosylates type IV pilin, while the other glycosylates multiple proteins (Harding et al., 2015). It appears that closely related bacterial *O*-OTases have evolved to target distinct substrates.

## 3 Diverse functions of neisserial glycoproteins

Altogether, over 50 glycoproteins have been identified in *N. gonorrhoeae* (Vik et al., 2009; Anonsen et al., 2012b; Hadjineophytou et al., 2024). The glycoproteins identified are mainly lipoproteins or transmembrane – domain containing proteins localized extracytoplasmically (as summarized in **Table 1**). A few of these glycoproteins have also been characterized in *N. meningitidis* (Stimson et al., 1995; Ku et al., 2009; Børud et al., 2010). However, immunoblotting with monoclonal glycan - specific antibodies shows a comparable repertoire of glycoproteins in both pathogenic species (Børud et al., 2010). Many diverse functions are associated with the glycoproteins, along with the type IV pilus biogenesis proteins, several glycoproteins are for instance involved in antimicrobial resistance or suggested as vaccine candidates for *N gonorrhoeae.*

**Table 1:** Glycoproteins identified in *N. gonorrhoeae* and *N. meningitidis*.

NGO	Alias	Protein	Description	Localization	References
Type IV biogenesis proteins - multiple roles					
–	NEIS0210, NMB0018	PilE	Type IV biogenesis protein, major glycoprotein in both *N. gonorrhoeae* and *N. meningitidis.* Pilus gonorrhea vaccine tested in men and women, antibody response was shown to pili from the vaccine strain and less to heterologous strains. The vaccine failed to protect against gonorrhea (Boslego et al., 1991).	Transmembrane domain, Periplasm, Cell surface	(Parge et al., 1995; Stimson et al., 1995; Vik et al., 2009; Anonsen et al., 2012b)
NGO0094	NEIS0408, NMB1812	PilQ	Type IV biogenesis protein Vaccine candidate for serogroup B *N. meningitidis*. Cross-species protection for *N. gonorrhoeae* (Haghi et al., 2012; Leduc et al., 2020). Resistance against tetracyclines through reduced influx (Unemo and Shafer, 2014).	Cell outer membrane, Membrane	(Anonsen et al., 2012b; Hadjineophytou et al., 2024)
Additional Type IV biogenesis proteins					
NGO0097	NEIS0411, NMB1809	PilN	Type IV biogenesis protein	Membrane, Transmembrane	(Hadjineophytou et al., 2024)
NGO0452	NEIS0827, NMB0886	PilH	Type IV biogenesis protein	Cell inner membrane, Cell membrane, Membrane	(Hadjineophytou et al., 2024)
NGO0453	NEIS0828, NMB0887	PilI	Type IV biogenesis protein	Membrane, Transmembrane	(Hadjineophytou et al., 2024)
NGO0454	NEIS0829, NMB0888	PilJ	Type IV biogenesis protein	Membrane	(Hadjineophytou et al., 2024)
NGO1177	NEIS1995, NMB2016	ComP	Minor pilin	Transmembrane	(Anonsen et al., 2012b)
NGO1441	NEIS0487, NMB0547	PilV	Minor pilin	Periplasmic protein	(Hadjineophytou et al., 2022)
Vaccine antigens					
NGO1043	NEIS2446,	Ag473	Lipoprotein. Meningococcal antigen Ag473 can elicit protective immune responses in mice (Chu et al., 2012). Gonococcal NGO1043 have low bactericidal activity against *N. gonorrhoeae* strains (Zhu et al., 2019)	Lipoprotein	(Vik et al., 2009; Anonsen et al., 2012b; Hadjineophytou et al., 2024)
NGO1205	NEIS0944, NMB0964	ZnuD	Zinc receptor/uptake component D. Putative TonB outer-membrane receptor protein. Antibodies detected in sera after meningococcal disease (Stork et al., 2010). Identified as candidate *N. gonorrhoeae* vaccine antigen in bioinformatic assessments (Baarda et al., 2021).	Cell outer membrane, Membrane	(Hadjineophytou et al., 2024)
NGO1225	NEIS1487, NMB1567	Mip	Macrophage Infectivity Potentiator Protein. Peptidylprolyl isomerase (EC:5.2.1.8). Surface-exposed and capable of inducing functional bactericidal antibodies against *N. gonorrhoeae* and *N. meningitidis* strains (Leuzzi et al., 2005; Humbert and Christodoulides, 2018).	Periplasmic protein	(Vik et al., 2009; Anonsen et al., 2012b; Hadjineophytou et al., 2024)
NGO1276	NEIS1549	AniA/NirK	Copper-containing nitrite reductase, essential for gonococci in oxygen-limiting conditions, elicit functional blocking antibodies against AniA in animal studies (Shewell et al., 2017). Identified as glycoprotein in *N. meningitidis*.	Cell outer membrane, Membrane	(Ku et al., 2009; Vik et al., 2009; Børud et al., 2010; Anonsen et al., 2012b; Hadjineophytou et al., 2024)
NGO1494	NEIS1689, NMC1689	PotF3	Putrescine binding periplasmic protein. Putative polyamine permease substrate-binding protein. Gonococcal OMV vaccine induced response in mice to PotF3 (Liu et al., 2017).	Binding Periplasmic Protein	(Vik et al., 2009; Anonsen et al., 2012b; Hadjineophytou et al., 2024)
NGO1577	NEIS1783, NMB0382	RmpM	Outer membrane protein class 4 RmpM antibodies are bactericidal against *N. meningitidis* (Rosenqvist et al., 1999; Williams et al., 2014).	Cell outer membrane, Membrane	(Hadjineophytou et al., 2024)
NGO1812	NEIS2020, NMB2039	PorB/PenB	Gonococcal PorB as a DNA-based vaccine generated anti-porin antibodies and induce both a Th1 and Th2 responses in mice (Zhu et al., 2005). PorB induce functional immune responses against MenB strains after 4CMenB vaccination (Viviani et al., 2023).	Cell outer membrane, Membrane	(Hadjineophytou et al., 2024)
NGO2139	NEIS1917, NMB1946	GNA1946, MetQ	Putative methionine binding component of an ABC transporter. Surface exposed lipoprotein, elicit bactericidal and functionally blocking mouse antibodies (Semchenko et al., 2017; Sikora et al., 2020).	Membrane	(Vik et al., 2009; Anonsen et al., 2012b; Hadjineophytou et al., 2024)
Antimicrobial resistance^1^					
NGO0099	NEIS0414, NMB1807	PonA	Penicillin binding protein 1; peptidoglycan glycosyltransferase. Resistance against penicillins.	Cell inner membrane, Cell membrane, Membrane	(Hadjineophytou et al., 2024)
NGO1364	NEIS1633, NMB1716	MtrD	MtrCDE efflux pump complex	Membrane	(Anonsen et al., 2012b; Hadjineophytou et al., 2024)
NGO1365	NEIS1634, NMB1715	MtrC	MtrCDE efflux pump complex	Cell inner membrane, Cell membrane, Membrane	(Anonsen et al., 2012b; Hadjineophytou et al., 2024)
NGO1439	NEIS0489, NMB0549	MacB	Macrolide-specific efflux pump protein; ABC transporter	Periplasmic protein	(Hadjineophytou et al., 2024)
NGO1440	NEIS0488, NMB0548	MacA	Macrolide-specific efflux pump protein; ABC transporter	Cell inner membrane, Cell membrane, Membrane	(Anonsen et al., 2012b; Hadjineophytou et al., 2024)
NGO1683	NEIS1853, NMB0318	FarA	Efflux pump protein, fatty acid resistance.	Membrane	(Hadjineophytou et al., 2024)
NGO1812	NEIS2020, NMB2039	PorB/PenB	Major outer membrane porin, reduce influx.	Cell outer membrane, Membrane	(Hadjineophytou et al., 2024)
Other glycoproteins					
NGO0016	NEIS0333, NMB1888	SecG	Preprotein translocase subunit SecG	Cell membrane, Membrane	(Hadjineophytou et al., 2024)
NGO0176	NEIS0536, NMB0594		Putative two-component system sensor kinase	Membrane, Transmembrane	(Hadjineophytou et al., 2024)
NGO0265	NEIS0643, NMB0692		Putative tetrapac protein	Membrane, Transmembrane	(Hadjineophytou et al., 2024)
NGO0372	NEIS0739, NMB0787		Putative ABC transporter, putative amino acid permease substrate-binding protein	Periplasmic protein	(Vik et al., 2009; Anonsen et al., 2012b; Hadjineophytou et al., 2024)
NGO0572	NEIS1270, NMB1332		Putative carboxy-terminal processing protease	Periplasmic protein	(Hadjineophytou et al., 2024)
NGO0994	NEIS1462, NMB1533	Laz	H.8 outer membrane protein	Cell outer membrane, Membrane	(Vik et al., 2009; Anonsen et al., 2012b)
NGO1237	NEIS1498, NMB1578	Sco	Lipoprotein	Periplasmic protein	(Vik et al., 2009; Anonsen et al., 2012b)
NGO1285	NEIS1556, NMB1642	NusA	Transcription elongation factor NusA	Cytoplasm	(Hadjineophytou et al., 2024)
NGO1320	NEIS1589, NMB1671		Putative paraquat-inducible protein B	Membrane	(Hadjineophytou et al., 2024)
NGO1328	NEIS1595, NMB1677	CycB	C-type cytochrome	Membrane, Transmembrane	(Vik et al., 2009; Anonsen et al., 2012b; Hadjineophytou et al., 2024)
NGO1371	NEIS1640, NMB1723	CcoP	Cytochrome c oxidase subunit III, cbb3-type cytochrome c oxidase subunit III	Periplasmic protein	(Vik et al., 2009; Anonsen et al., 2012b; Hadjineophytou et al., 2024)
NGO1393	NEIS0596, NMB0652	MafA2	Putative secretion of MafB polymorphic toxins	Cell outer membrane, Membrane	(Hadjineophytou et al., 2024)
NGO1584	NEIS1789, NMC1789	mafAMGI-1	Putative secretion of MafB polymorphic toxins		(Hadjineophytou et al., 2024)
NGO1415	NEIS0508, NMB0567		Na(+)-translocating NADH-quinone reductase subunit C	Cell inner membrane, Cell membrane, Membrane	(Hadjineophytou et al., 2024)
NGO1492	NEIS1687, NMB0464		Outer membrane phospholipase A precursor (ec 3.1.1.32)	Cell outer membrane, Membrane	(Hadjineophytou et al., 2024)
NGO1717	NEIS0273, NMB0278	DsbA1	Thiol:disulphide interchange protein encodes DsbA1; oxidoreductase	Periplasmic protein	(Vik et al., 2009; Anonsen et al., 2012b; Hadjineophytou et al., 2024)
NGO1769	NEIS2721		Cytochrome-c peroxidase	Cytochrome C peroxidase	(Anonsen et al., 2012b; Hadjineophytou et al., 2024)
NGO1800	NEIS0174, NMB0183		Putative inner membrane protease	Transmembrane	(Hadjineophytou et al., 2024)
NGO2002	NEIS2053, NMB2074		Putative periplasmic protein	Periplasmic protein	(Hadjineophytou et al., 2024)
NGO2092	NEIS1964, NMB1989		Putative membrane transport solute-binding protein	Periplasmic protein	(Hadjineophytou et al., 2024)
NGO2094	NEIS1949, NMB1973		Co-chaperonin GroES	Cytoplasm	(Hadjineophytou et al., 2024)
Hypothetical proteins					
NGO0360	NEIS0731, NMB0778		Hypothetical protein	Membrane, Transmembrane	(Anonsen et al., 2012b; Hadjineophytou et al., 2024)
NGO0561	NEIS1281, NMB1345		Hypothetical protein	–	(Hadjineophytou et al., 2024)
NGO0666	NEIS1287, NMB1352		Hypothetical protein	–	(Hadjineophytou et al., 2024)
NGO0983	NEIS1452, NMB1523		Hypothetical protein	Cell outer membrane, Membrane	(Anonsen et al., 2012b)
NGO1067	–		Hypothetical protein	Cell outer membrane, Membrane	(Hadjineophytou et al., 2024)
NGO1972	–		Hypothetical protein	Cell outer membrane, Membrane	(Hadjineophytou et al., 2024)
NGO06725	–		Hypothetical protein	Cell outer membrane, Membrane	(Hadjineophytou et al., 2024)
NGO10270	–		Hypothetical protein	Periplasmic protein	(Hadjineophytou et al., 2024)

^1^Proteins involved in antimicrobial resistance mechanisms was obtained from (Unemo and Shafer, 2014).

Several gonococcal proteins that elicit bactericidal or functional blocking antibodies have been suggested as vaccine candidates are known glycoproteins (**Table 1**), i.e PilE (Boslego et al., 1991), Mip (Leuzzi et al., 2005; Humbert and Christodoulides, 2018), AniA (Shewell et al., 2017), PilQ (Haghi et al., 2012; Leduc et al., 2020), MetQ (Semchenko et al., 2017; Sikora et al., 2020), PorB (Zhu et al., 2005), ZnuD (Stork et al., 2010; Baarda et al., 2021) and PotF3 (Liu et al., 2017). Some vaccines against serogroup B meningococcal (MenB) disease have been based on outer membrane vesicles (OMVs) that include a broad range of OMV proteins, and the 4CMenB also contains recombinant protein antigens to increase the protection across diverse MenB strains (Holst et al., 2009; Micoli and Maclennan, 2020; Viviani et al., 2022). Although not the major antigens in these vaccines, there are numerous antigens that contribute to the protective effect against MenB strains that are known glycoproteins; PorB (Viviani et al., 2023), PilQ (Haghi et al., 2012), Ag473 (Chu et al., 2012), RmpM (Rosenqvist et al., 1999; Williams et al., 2014), and Mip (Humbert and Christodoulides, 2018) (**Table 1**). Given the fact that glycans impact on the immunogenicity, as shown for the PilE protein (Børud et al., 2010), glycan function and diversity may need to be considered when including glycoproteins in future vaccines. As such, the complete glycoproteomes, potential intra- and interspecies differences should be investigated to understand the full, biological significance of glycosylation.

Emergence of multidrug resistant *N. gonorrhoeae* is making treatment more difficult, and the risk of untreatable disease represents a major global public health concern. It is therefore important to understand the molecular and phenotypic mechanisms involved. The recent identification of additional glycoproteins revealed that several of those are involved in antimicrobial resistance mechanisms (**Table 1**) (Hadjineophytou et al., 2024). The detected glycoproteins are involved in efflux (MtrC, MtrD, MacA, MacB, FarA) and influx (PorB) of antimicrobials. The MtrCDE efflux pump exports diverse hydrophobic antimicrobials (macrolides, penicillin, ciprofloxacin and tetracyclin), while the MacAB efflux pump export macrolides and FarAB efflux pump export cationic antimicrobial peptides and long-chain fatty acids (Unemo and Shafer, 2014). The glycoproteins PorB/PenB, PilQ and PonA are involved in resistant to penicillin through different mechanisms (Unemo and Shafer, 2014). The potential influence of glycosylation of these proteins on antimicrobial resistance through molecular fine tuning or altering activity remains to be considered.

By examining known glycopeptides in *N. gonorrhoeae* MS11 replicates using Data-Independent Acquisition (DIA) analysis, different glycosylation occupancy frequencies were found without affecting the protein abundance (Hadjineophytou et al., 2024). This study also discovered that the glycan occupancy on glycoproteins was often low. There are few if any studies addressing potential regulation of protein glycosylation in *Neisseria.* However, information about regulation mechanisms could impact on our understanding of protein glycosylation; both on the level of individual glycoproteins and their functions, as well as on our understanding of glycan diversity and immune escape mechanisms.

## 4 *Neisseria* protein glycans are immunogenic

The surfaces of all cells in nature exhibit taxon-, species-, and cell-type-specific characteristics in their intricate layer of glycans (Varki, 2011). Serving as major components of outermost surface molecules, glycans play crucial roles in many processes. In general, this includes host-pathogen interactions, immunological recognition and activation, and differentiation between self and nonself through a sophisticated array of pathways and mechanisms. Microbes often exploit host glycans as targets for cellular binding and tissue invasion and some have developed mechanisms of glycan mimicry or extensive glycan variability to elude the host response. Additionally, microbial glycans can serve as a protective glycan shield by hindering access to underlying protein epitopes as reviewed elsewhere (Zhou and Cobb, 2021).

The adaptive immune system in vertebrate organisms primarily operates through the recognition of foreign peptide sequences. These sequences are directly acknowledged by the B cell surface Ig receptor and are also loaded into the grooves of major histocompatibility receptors for presentation to specific T-cell receptors (Hoogeboom and Tolar, 2016). When the peptide carries a small glycan, this component can introduce novel specificity to recognition of the peptide (Varki, 2017). In fact, PilE-associated glycans are immunogenic, as well as antigenically variable when expressed in different protein glycosylation (*pgl*) gene backgrounds (Børud et al., 2010).

Recently, we suggested that meningococcal carriage and disease stimulate production of antibodies against different neisserial glycoforms in humans. We found that most of the Ethiopian patients (83%) infected with serogroup A ST-7 *N. meningitidis* and a proportion of the control group (24%) without any history of meningococcal disease, had antibodies against neisserial protein glycan antigens (Naess et al., 2023). In addition, by using a bactericidal assay comparing a wild type meningococcal A strain and a glycosylation-null variant strain, it was shown that the protein glycan antigens may protect against bactericidal killing by antibodies in Ethiopian patient sera, possibly by masking protein epitopes important for bactericidal killing and thus protection against meningococcal disease (Naess et al., 2023). The pilin glycan may be a target for anti-Gal IgA antibodies in natural human serum that has been reported to bind to meningococcal pili and block complement-mediated lysis (Hamadeh et al., 1995).

There are several successful vaccines targeting capsular polysaccharides in bacteria, such as the highly immunogenic and efficient conjugated vaccines against *Streptococcus pneumoniae, Haemophilus influenzae* type b, and *N. meningitidis* capsular polysaccharides for serogroups A, C, W and Y. These carbohydrate-based vaccines are made by conjugation of extracted polysaccharides to different carrier proteins (Lindberg, 1999). Bacterial protein glycosylation pathways can also be exploited in glyco-engineering to create glycoconjugates by using oligosaccharyltransferases to generate bacterial vaccines (Feldman et al., 2005; Price et al., 2016). To our knowledge however, the only engineered vaccines targeting glycan antigens of glycoproteins involve the *Campylobacter jejuni N*-glycan developed for chickens. Two different glycoconjugate vaccines were constructed; the *N*-glycan attached to a protein carrier or fused to the *Escherichia coli* lipopolysaccharide-core. Vaccination of chickens with either showed reduction in *C. jejuni* colonization and induced glycan-specific IgY responses (Nothaft and Szymanski, 2010). Transplantation of microbiota in combination with vaccination further increased the vaccine-induced antigen-specific IgY responses (Nothaft et al., 2021). It is worth noting in this context that the majority of the *N*-glycan found in *C. jejuni* is found in its free oligosaccharide form (Nothaft et al., 2009).

## 5 Glycosylation pathway and *pgl* gene content

**Figure 1:**
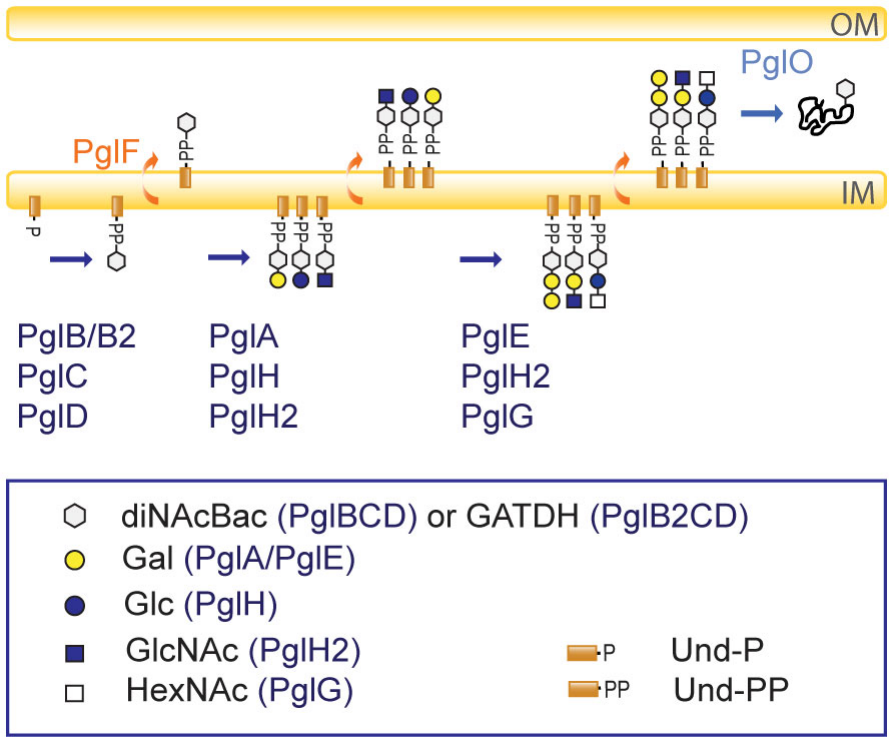
Simplified overview of the *O*-linked protein glycosylation pathway in *N. gonorrhoeae* and *N. meningitidis*. PglB/PglB2, PglC, and PglD synthesizes the undecaprenyl diphosphate (Und-PP) -linked monosaccharides. The PglB variant synthesizes the monosaccharide N, N′-diacetylbacillosamine (diNAcBac), while the PglB2 variant is responsible for synthesis of glyceramido-acetamido trideoxyhexose (GATDH). PglF translocates the synthesized glycans into the periplasm continuously. The glycosyltransferases PglA, PglH, PglH2 adds galactose (Gal), glucose (Glc) or N-acetylglucosamine (GlcNAc), respectively, to the monosaccharides. The glycosyltransferases PglE, PglH2 and PglG add galactose (Gal), glucose, N-acetylglucosamine (GlcNAc) or an *N*-acetylhexosamine (HexNAc), respectively, to the disaccharides. The *O*-OTase PglO transfers the glycans onto proteins in the periplasm. O-acetylation is mediated by the acetyltransferase PglI (not shown here, see **Figure 2** for further details on acetylation). The figure legend shows the symbols used with the involved Pgl protein in parenthesis. OM, outer membrane; IM, inner membrane. All glycans in the figure can have either diNAcBac or GATDH as the first sugar and are therefore colored white (generic) according to the Symbol Nomenclature for Glycans (https://www.ncbi.nlm.nih.gov/glycans/snfg.html).

**Figure 2:**
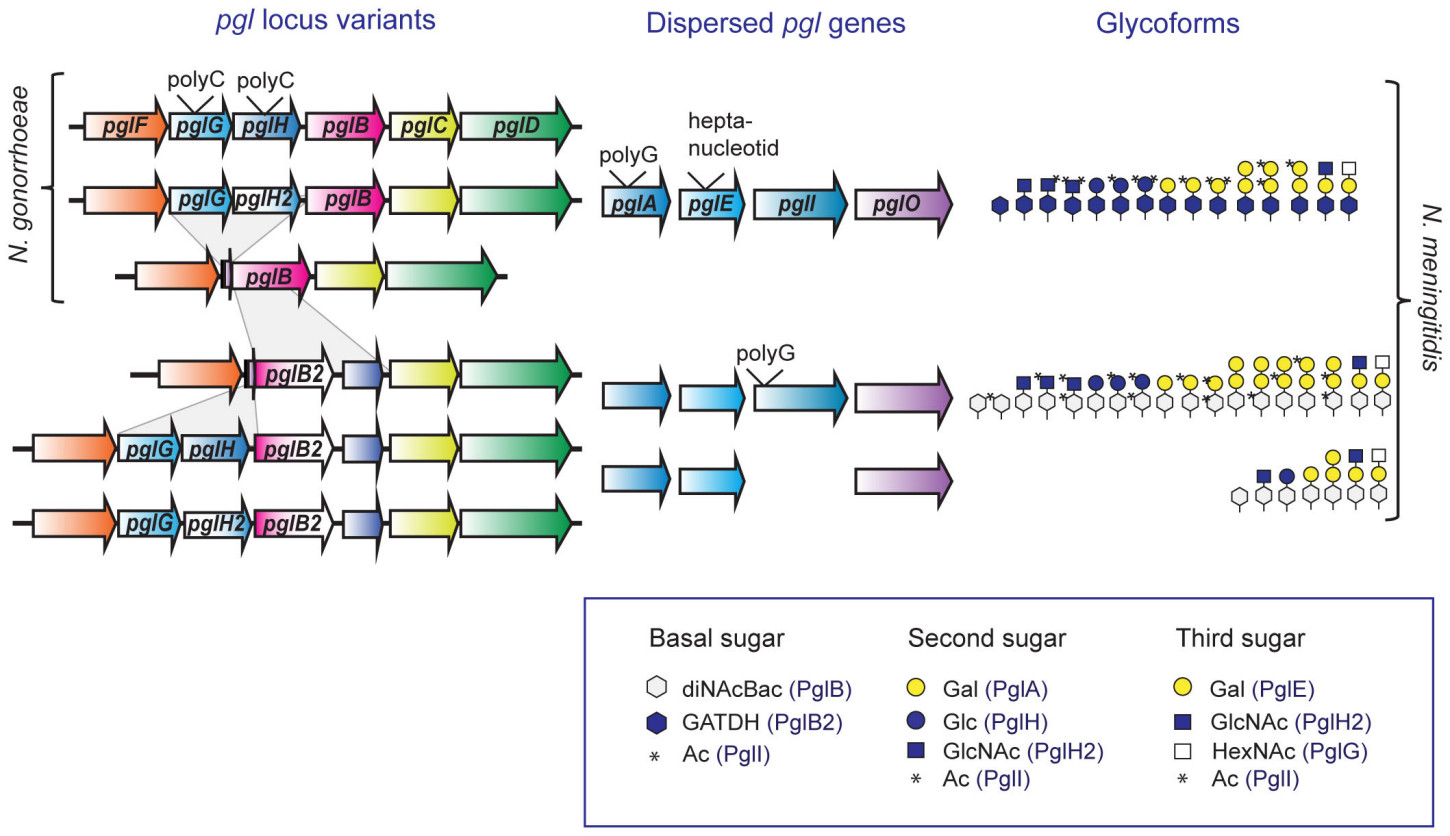
Protein glycosylation gene variants and glycoform outcome in pathogenic *Neisseria*. An overview of the *pgl* genes present in different *pgl* loci variants and the currently known glycoforms synthesized by *N. gonorrhoeae* and *N. meningitidis* carrying these gene combinations. The *pgl* genes *pglF, pglG, pglH, pglB/B2, pglC*, and *pglD* are linked together in the *pgl* locus, whereas *pglA, pglE, pglI* and *pglO* are dispersed elsewhere. The two major recombination events in the *pgl* loci are shown, the *pglB2-ORF8* insertion and the *pglG-pglH* deletion, see main text for more details. The genes *pglA, pglE*, *pglG* and *pglH/H2* contains phase variable tracts as shown, although there are also non-phase variable allele variants for these genes. The *pglI* can be missing or phase variable in *N. meningitidis*, but non-phase variable in *N. gonorrhoeae*. The figure legend shows the symbols used with the involved Pgl protein in parenthesis. diNAcBac, N, N′-diacetylbacillosamine; GATDH, glyceramido-acetamido trideoxyhexose; Gal, galactose; Glc, glucose; HexNAc, *N*-acetylhexosamine; GlcNAc, N-acetylglucosamine; Ac, acetyl. Contrary to the generic white color of the first sugar in **Figure 1**, we here used white for GATDH glycoform variants and blue for diNAcBac variants according to the Symbol Nomenclature for Glycans (https://www.ncbi.nlm.nih.gov/glycans/snfg.html).

An overview of the currently known glycosylation pathways in pathogenic *Neisseria* is shown in **Figure 1**. The *pgl* core locus products function in the synthesis of Und-PP monosaccharides on the cytoplasmic face of the inner membrane (PglB/B2, PglC, PglD) and translocation into the periplasm (PglF). PglB is a bifunctional protein (acetyltransferase/phosphorglycosyl-transferase) responsible for synthesis of N,N′-diacetyl-bacillosamine (diNAcBac), while the variant PglB2 (ATP grasp/phosphorglycosyl-transferase) is responsible for synthesis of glyceramido acetamido trideoxyhexose (GATDH) (Chamot-Rooke et al., 2007). PglA and PglE are galactosyltransferases that elaborate the mono- and disaccharide, respectively, by adding galactose (Gal) (Aas et al., 2007). PglH/H2 is a glycosyltransferase that generate glucose (Glc) or N-acetylglucosamine (GlcNAc)-containing disaccharides, respectively. Alternatively, PglH2 can extend the PglA disaccharide to a GlcNAc terminating trisaccharide (Børud et al., 2011, 2014). It has also recently been shown that *pglG* alleles from *N. meningitidis* are associated with incorporation of an N-acetylhexosamine (HexNAc) at the third position (Wang et al., 2021). Diminished level of pilin-linked glycan in *N. gonorrhoeae pglF* mutants, together with PilF homology to ABC transporter-type flippases implies a role in the translocation of the Und-PP-linked glycan across the periplasmic membrane (Aas et al., 2007; Hartley et al., 2011). PglF continuously translocates the glycans into the periplasm during synthesis and cause some of the observed microheterogeneity. PglO (aka PglL) is the *O*-OTase that transfers the glycan to protein substrates (Power et al., 2006; Aas et al., 2007). A recent study by Hadjineophytou and colleagues demonstrate how different neisserial PglOs have distinct protein targeting activities (Hadjineophytou et al., 2022). In addition, neisserial glycoforms can be further modified via *O*-acetylation mediated by the acetyltransferase PglI (Aas et al., 2007; Børud et al., 2014). Altogether, 31 distinct glycoforms have been identified in pathogenic *Neisseria;* all of these are found within *N. meningitidis* while *N. gonorrhoeae* (lacking *pglB2*) is only capable of synthesizing 15 different glycoforms (**Figure 2**) (Anonsen et al., 2017). The high glycan diversity reveals that the enzymes acting downstream of the synthesis of the Und-PP-linked saccharides (PglB/PglB2-associated glycosyl-1-phosphate transferase, PglF flippase, and PglO *O*-OTase) retain relaxed donor specificity.

Genomic analyses of *Neisseria* species have shown variation due to horizontal gene transfer, both at the level of sequence diversity and gene content variation (Kong et al., 2013). Within neisserial *pgl* genes, there are both intra-species and inter-species genetic variation (**Figure 2**). There are two major polymorphisms at the core *pgl* locus; the variable presence of *pglG* and *pglH*, and the mutually exclusive presence of *pglB* and *pglB2*. While *pglB*, *pglG* and *pglH* are found in both pathogenic species, *pglB2* are only found within *N. meningitidis* and commensal *Neisseria*. The *pglB2*-ORF8 fragment have been inserted into the *pglB* gene, and both PglB and PglB2 have the same N-terminal domain with phosphoglycosyl-transferase activity (Kahler et al., 2001; Power et al., 2003). Neisserial strains carrying deletions of *pglG* and *pglH/H2* still bear conserved traces of the 5′ end of *pglG* and 3′ end of *pglH*; suggesting that the intact state is ancestral and that a deletion event likely occurred once and then spread through the populations (Børud et al., 2011). The presence of *pglG-pglH* was reported in 67% of strains in a predominantly meningococcal strain collection (Power et al., 2003) and in our previous study to 81% of gonococcal strains, 65% of meningococcal strains, and 94% of commensal strains (Børud et al., 2011). In addition, we have reported that polymorphism also exist at gene level as described for *pglH/pglH2* where a single non-synonymous mutation accounts for the glycoform switch from Glc to GlcNAc (Børud et al., 2014).

The glycosyltransferase genes *pglA* and *pglE* are not linked to the *pgl* loci or one another, and they are present in all pathogenic strains while absent in most commensal species. Hadjineophytou and colleagues suggest that the terminating galactose residues may have favorable function for the pathogenic *Neisseria*, for example in regard to recognition of the immune system, glycoproteins abundance or properties, or with variable metabolic costs (Hadjineophytou et al., 2022). The authors also proposed that the elaboration of monosaccharides followed two different pathways; the *pglA-pglE* pathway whose products add galactose to generate di- and trisaccharides and the pathway involving the *pglG-pglH/H2* insertion resulting in glucose- and/or GlcNAc -containing glycans (Hadjineophytou et al., 2022). Furthermore, as both pathways are active in several neisserial strains, competition and redundancy may be responsible for amorphic and hypomorphic *pglA* and *pglH* alleles, as well as the *pglG-pglH* deletion (Børud et al., 2011; Johannessen et al., 2012; Hadjineophytou et al., 2022).

The glycan diversity appears to be even more complex in commensals than the two pathogenic species (Hadjineophytou et al., 2019). Since variant commensal *pgl* genes could be introduced by homologous recombination, this could significantly contribute to further glycan diversity in pathogenic *Neisseria.* In support of this, Jen et al. identified disaccharides and trisaccharides with an uncharacterized basal sugar in *N. meningitidis* serogroup A ST-2859 (Jen et al., 2023).

## 6 Glycan diversity and immune escape

Bacterial genetic variation is important to avoid the adaptive immune response, and extensive allelic diversity in the genus *Neisseria* has been described, particularly in genes under antigenic selection pressure. The mechanism of phase variation involves reversible, spontaneous changes in the expression of specific genes, and it is facilitated by a phenomenon known as slippage during DNA replication within simple DNA repeats, either in the promoters or within the open reading frames. Slippage and variations in the number of repetitive DNA sequences leads to altered gene expression or frameshift mutations. This dynamic process allows bacteria to rapidly switch on or off the expression of certain genes; promoting adaptability and facilitating survival in response to changing environmental pressures. In pathogenic *Neisseria* more than 100 phase variable genes was postulated (Snyder et al., 2001), including several *pgl* genes that have mononucleotide or polynucleotide repeats tracts (Power et al., 2003). In a more recent analysis using a substantially higher number of genomes, the number of phase variable genes was reduced to a maximum of 47 and 54 per genome of *N. meningitidis* and *N. gonorrhoeae*, respectively (Wanford et al., 2018).

Slipped-strand mispairing-induced phase variation occurs repeatedly, creating heterogeneity within the population, and manifesting in a diverse range of phenotypes. It has been shown that a single isolate has the capacity to express up to 15 different glycans by combination of glycosyltransferases and the *O*-acetylase (Anonsen et al., 2017; Wang et al., 2021). The phase variable expression of *pglA, pglE*, *pglG pglH/H2* and *pglI* genes thus results in intrastrain glycan length variation (Power et al., 2003; Aas et al., 2007; Power et al., 2007; Børud et al., 2011; Anonsen et al., 2017; Wang et al., 2021). Consequently, the population consistently harbors variants pre-adapted to various environmental conditions and allowing them to withstand genetic bottlenecks by adjusting gene expression levels accordingly.

Protein glycan variation mediated by phase variation is unique to *Neisseria*, and the frequency of phase variable *pgl* genes appear to be higher in *N. meningitidis* than *N. gonorrhoeae* and is only found in the closely related commensals *Neisseria lactamica* and *Neisseria polysaccharea* (Børud et al., 2011; Wanford et al., 2018; Wang et al., 2021). For instance, the *O*-acetylation on oligosaccharides is subject to the phase-variable expression of *pglI* in *N. meningitidis*, while in *N. gonorrhoeae pglI* is non-phase variable and always expressed. As such, on–off modulation of PglI expression in *N. meningitidis* results in alterations between oligosaccharides with or without acetylation modification and thus increases the basic repertoires of oligosaccharides (Anonsen et al., 2017). Moreover, while *pglI* is present in all *N. gonorrhoeae*, it is absent in most commensals and some sequence types of *N. meningitidis*. Also, the *pglA* and *pglE* glycosyltransferase genes are absent in nearly all commensals, except for *N. lactamica* and *N. polysaccharea* (Børud et al., 2011; Hadjineophytou et al., 2019).

Within-host evolution involves the adaptation of a bacterial pathogen to colonization within a specific host. In *N. meningitidis*, within-host evolution is proposed to occur during the initial colonization phases by favoring variants that evade host immunity and colonize the epithelium and without necessarily selecting for increased fitness in invasive contexts. In one study, during an accidental human passage it was shown that *pglA* and *pglI* were turned off in the isolate retrieved from blood culture and not in the laboratory parental strain. Such simple sequence repeats tract variation led to altered glycoform expression, from diNAcBac-Gal-AcGal to diNAcBac-Glc, after human passage. The authors proposed that such changes could confer an advantage to the bacteria to escape the immune system (Omer et al., 2011).

Klughammer and colleagues examining paired isolates from throat swabs and blood culture in four patients with invasive meningococcal disease and detected phase variation of *pilC1* and *pglI*, along with gene conversion events in *pilE* (Klughammer et al., 2017). In a recent study, sequence analysis revealed within-host genetic changes in paired meningococcal carriage isolates from Ethiopia when analyzing 50 carriers with samples taken six to nine weeks apart (Bårnes et al., 2017). Among the most frequently altered genes were genes belonging to the restriction/modification systems, opacity proteins and genes involved in pilin antigenic variation and protein glycosylation (*pglG, pglH* and *pglI)* (Bårnes et al., 2017). These *pgl* genes showed phase variable differences resulting in on-off expression within the paired isolates; ranging from 60% of the pairs for *pglE* and 20% for *pglA* (Børud et al., 2018). Similarly, in another study by Mustapha and colleagues comparing paired carriage isolates from 188 individuals, the most frequently altered genes were *pilE*, the *opa* loci, and the *modA12* methyltransferase genes (Mustapha et al., 2021). Additionally, the phase variable *pgl* genes *pglA, pglE, pglH and pglI* exhibited high variability, and also a subset of genes underwent frequent microevolution during transmission but reached fixation during persistent carriage, including pilus biogenesis (*pilH, pilT, pilU* and *pilQ*) and glycosylation genes (*pglD*) (Mustapha et al., 2021). A comparable genetic pattern was noted in a controlled human infection study involving *N. lactamica* (Pandey et al., 2018). The study revealed that among *hpuA, fetA*, and *hsdS*, *pilE* and pilin glycosyltransferase genes *(pglA and pglH)* exhibited the highest variability during a one-month carriage period (Pandey et al., 2018).

There are also cases where homologous recombination within *pgl* genes results in permanent change of expressed glycans. One study compared genomes of serogroup Y, ST-23 clonal complex, and hypothesized that emergence of a late strain type was primarily due to antigenic changes that allowed escape from population immunity. Among the differences was a recombination event in the *pgl* loci exchanging *pglB* in the early strain type with *pglB2* in the late strain type (Krauland et al., 2012). The phenotypic consequences for replacement of the *pglB* allele with *pglB2*, is synthesis of GATDH - based glycoforms instead of diNAcBac glycoforms (Chamot-Rooke et al., 2007; Børud et al., 2010). The same recombination event exchanging *pglB* with *pglB2* was observed in two of 37 N*. meningitidis* serogroup A ST-7 isolates (Naess et al., 2023).

Another study detected sequence type specific homologous recombination within the *pgl* loci through genomic analysis of 100 isolates representing the clonal replacement of the hyper virulent serogroup A *N. meningitidis* clone ST-7 with the ST-2859 descendant clone. The authors suggested that this emphasized the role of protein glycosylation diversity in immune evasion (Lamelas et al., 2014). Our recent genome analysis of *N. meningitidis* serogroup A ST-7 isolates identified an IS element within *pglH*, and the *pgl* loci homolog recombination described by Lamelas and colleagues thus replaced the IS - disrupted *pglH* and likely led to increased glycan variability in ST-2859 compared to ST-7 isolates (Naess et al., 2023).

## 7 Concluding Remarks

In conclusion, glycosylation undoubtedly impacts on the antigenicity and immunogenicity of proteins, especially for the abundant and surface exposed pilin that has been extensively studied. As summarized above, several studies suggest that *N. meningitidis* evade the immune system by changing their protein glycan structures (Krauland et al., 2012; Lamelas et al., 2014; Gault et al., 2015). However, further studies are essential to confirm this hypothesis and to explore the mechanisms and potential differences in pathogenic and commensal *Neisseria*.

Understanding the diversity and biological role(s) of glycosylation employed by *Neisseria* can be crucial for developing effective vaccines and treatments, especially for *N. gonorrhoeae* where antimicrobial resistance is high and vaccine development has been difficult. Absence of natural protection after repeated gonorrhea infections or in previous vaccine attempts must, to some degree, be attributable to the high antigenically variable surface antigens in *N. gonorrhoeae.* A number of potential antigens are being considered and the general consensus is that a successful vaccine will incorporate multiple antigens. Attempts to identify conserved antigens through comparative genomics are ongoing but this approach has limitations because they overlook those whose structural features not linearly templated within genome sequences such as PTMs. Researchers are actively studying potential targets for intervention and to design strategies that can counteract the immune evasion tactics of these bacteria.

## Author contributions

BB: Writing – review & editing, Writing – original draft. MK: Writing – review & editing, Writing – original draft.
